# Predicting the Hamstring Graft Size for ACL Reconstruction Using a 3D Tendon Model in Preoperative MRI

**DOI:** 10.3390/jcm14062128

**Published:** 2025-03-20

**Authors:** Andreas Frodl, Moritz Mayr, Markus Siegel, Hans Meine, Elham Taghizadeh, Sebastian Bendak, Hagen Schmal, Kaywan Izadpanah

**Affiliations:** 1 Department of Orthopedics and Traumatology, Freiburg University Hospital, 79106 Freiburg, Germany; 2 Fraunhofer Institute for Digital Medicine MEVIS, Max-von-Laue-Str. 2, 28359 Bremen, Germany; 3 Department of Orthopedic Surgery, University Hospital Odense, Sdr. Boulevard 29, 5000 Odense, Denmark

**Keywords:** anterior cruciate ligament, Arthroscopic surgery, magnetic resonance imaging, graft size

## Abstract

**Background:** Rupture of the ACL is a common injury among men and women athletes. While planning the surgical ACL reconstruction procedure, the eventual graft’s diameter is extremely important. Many parameters are therefore evaluated pre-surgery to ensure access to reliable data for estimating the graft diameter. Considering this, magnetic resonance imaging (MRI), particularly qualitative analyses of the hamstring tendons, offers a promising approach. **Methods:** In a retrospective analysis, we carried out 3D segmentation of the gracilis (GT) and semitendinosus tendon (ST) utilizing MRI with varying slice thicknesses and field strengths. The cross-sectional area (CSA) was calculated on different levels (by relying on the models we had thus created) to generate a mean of CSA with six specific segments. We then correlated the mean CSA with the diameter of the graft measured during surgery. **Results:** A total of 32 patients were included (12 female, 20 male) in this retrospective analysis. We observed the largest CSA in segment 10 mm–0 (16.8 ± 6.1) with differences between men and women. The graft size and tendon diameter correlated significantly in all segments throughout our study cohort. The strongest correlation was apparent in the segment 10 mm–0 (r = 0.552). **Conclusions:** MRI-based 3D segmentation and the STGT CSA represent a reliable method for estimating preoperatively a quadrupled hamstring graft diameter. The 10 mm–0 mm segment above the joint line showed a strong correlation, making it an ideal reference for graft planning.

## 1. Introduction

Anterior cruciate ligament (ACL) rupture is a common injury among athletes and recreational sports enthusiasts, with women experiencing a threefold higher incidence of ACL injuries [1]. Surgeons have several options for harvesting and using grafts for surgical reconstruction. In addition to patellar and quadriceps tendons, hamstring tendons are frequently used as autologous grafts [2,3]. However, problems can arise during graft harvesting, as can postoperative complications if the graft diameter is insufficient [4,5]. For example, the risk of ACL re-rupture increases if there is insufficient graft strength [6].

To anticipate such challenges, efforts are being made to develop reliable methods for preoperative assessments, particularly of hamstring grafts. Several parameters have been investigated to facilitate such evaluations. These include anthropometric factors such as height, weight, BMI, and gender, with recent studies focusing on MRI diagnostics, particularly the cross-sectional area (CSA) of the semitendinosus (ST) and gracilis (GT) tendons [7,8,9,10]. However, these measurements have typically been taken at a single reference height. As these tendons reveal varying shapes along their course—from round to oval—results can differ depending on the measurement height, thus complicating a reliable evaluation [7,11]. However, the use of artificial intelligence and deep learning processes now allows for the creation of increasingly precise models and more reliable segmentation of MRI data [12,13,14]. 

We hypothesized that there is a significant correlation between the hamstring graft size used for ACL replacement and the cross-sectional area (CSA) of the semitendinosus (ST) and gracilis tendons (GT) at different levels.

## 2. Materials and Methods

### 2.1. Patients

This retrospective study was conducted at the medical center of Freiburg University following the STROBE guidelines and in accordance with the Declaration of Helsinki. Approval was obtained from our institutional review board (447/19). The study was registered in the German Trials Register (DRKS 00031502). Thirty-two patients undergoing a single bundle using an autologous ipsilateral quadrupled ham-string graft (semitendinosus tendon and gracilis tendon each doubled) were included in this observational analysis. For inclusion, patients had to meet these criteria: age between 14 and 60 years, ACL reconstruction with an autologous ipsilateral quadrupled hamstring graft (STGT), complete preoperative MRI dataset of the injured knee joint, and an intraoperatively documented graft diameter. Excluded patients were those having already undergone ipsilateral ACL reconstruction, having had an acute or chronic ipsilateral hamstring injury, as well as those with missing or corrupted digital MRI data. Relying on the electronic database of each clinical center, datasets were screened retrospectively from January 2016 to December 2017 using the ICD-10 Code for ACL rupture and treated by one surgeon. Thus, 121 patients were identified; 51 patients were treated by different surgeons and had to be excluded. From the remaining 70 patients, only 32 complete records met our inclusion criteria for further analysis. From these individuals, we analyzed the MRI data and collected basic demographic information (age, sex). Relative influencing factors such as BMI, muscle–fat ratio, and body height were not recorded in this exploratory setting.

**Figure 1:**
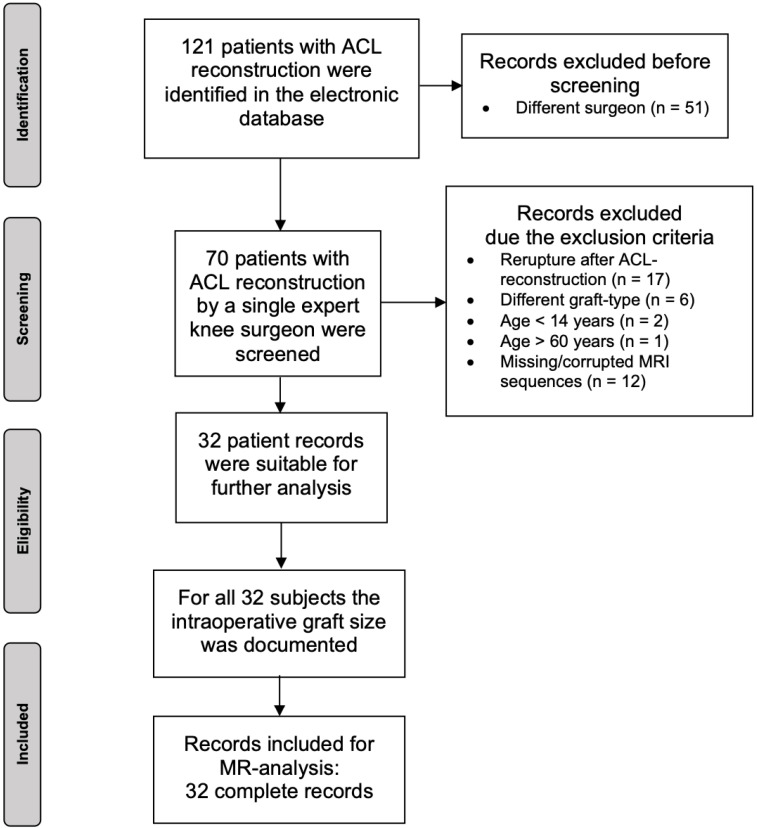
Flow chart of the patient-selection process.

A detailed overview of this inclusion process is found in Figure 1.

### 2.2. Methodological Approach

**Figure 2:**
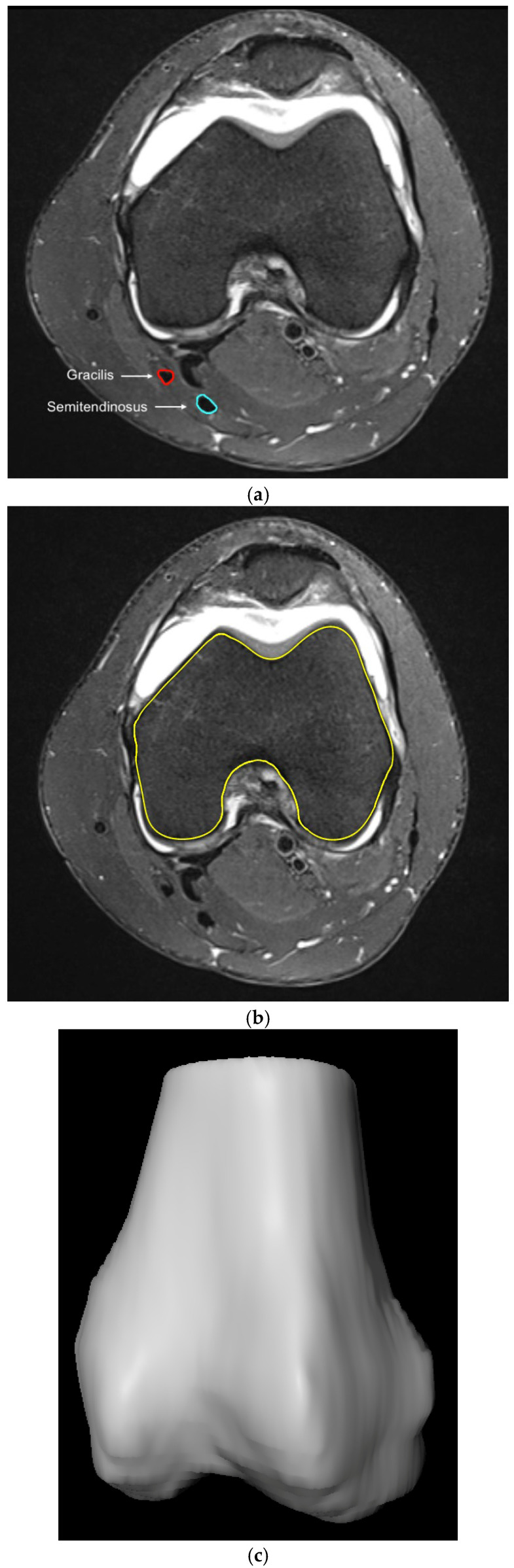
(**a**). Digital segmentation of the gracilis (red) and semitendonosus (light blue) tendon. (**b**). Digital segmentation of the femoral bone (yellow). (**c**). 3D model of the femoral bone visualized by SoDa cartilage analysis.

MRI images were imported to SoDa-CartilageAnalysis (MeVis Medical Solutions AG & Fraunhofer MEVIS, Bremen, Germany) and the semitendinosus and gracilis tendons were marked with the freehand function using axial images. The femoral outline was also drawn manually in all axial layers. The datasets generated through the segmentation process of the femur and tendons were then used for three-dimensional reconstruction and subsequent analysis generated by the MeVisLab platform and ShowACLContactSurface software (Ver. 1.1, MeVis Medical Solutions AG, Bremen, Germany) (Figure 2a–c).

**Figure 3:**
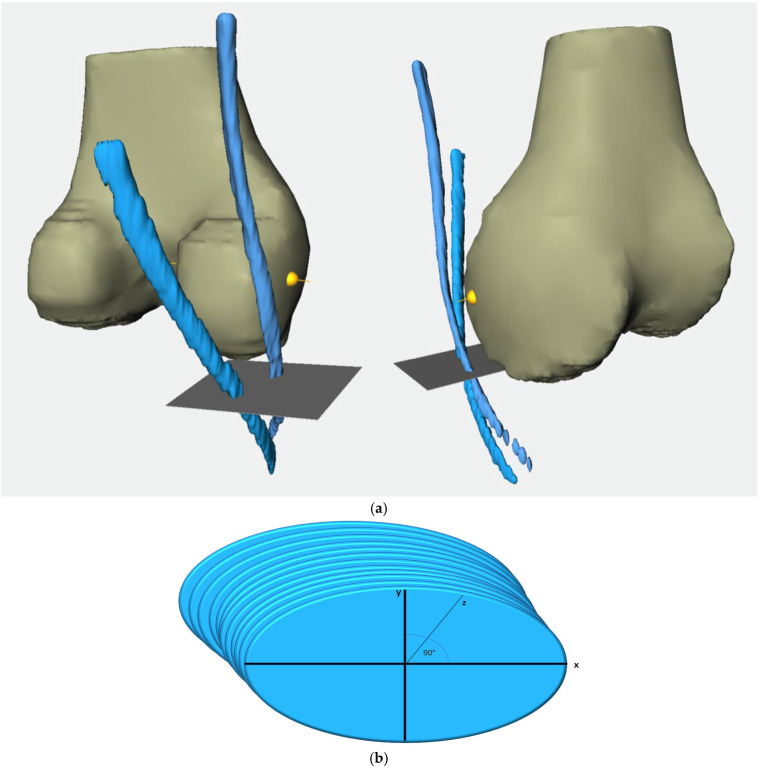
(**a**). 3D representation of the reconstructed semitendinosus and gracilis tendons (blue strands) and the femur using ShowACLContactSurface. The gray plane corresponds to the reference level of the joint line, while the yellow line represents the reference level WID. Left: view from dorsomedial, right: view from ventromedial. (**b**). Exemplary view of segmentated CSA with drawn main axis.

Relying on the segmented femur bone, we established a coordinate system where the projection of the femur segmentation served as the third spatial axis (*z*-axis) and the lowest segmented femur layer was considered as the joint line. To align the femoral shaft axis with the correct MRI image axis, we first determined the center point of each segmented femur layer. These center points were then connected by a line which then served to align the femoral axis and to determine the widest intercondylar diameter (WID) [15]. Lastly, the previously segmented contours of the semitendinosus tendon (ST) and gracilis tendon (GT) were incorporated within the coordinate system and aligned with the femoral axis. (Figure 3a,b)

To calculate the cross-sectional areas of the tendons, we inserted an *x*-axis and *y*-axis, positioned at a 90° angle to each other into each segmented layer. The cross-sectional area was determined in all marked layers in this manner. The subsequent division of the tendon course into clearly defined segments enabled us to calculate an average value for each segment, which was then derived from all area measurements across the axial MRI slices within a specified tendon section. Segmentation into six specific sections served to provide a standardized framework for analyzing and comparing the tendons of patients. The height of the joint line (JL) was set as point 0, as it served as the origin point for the subsequently created three-dimensional coordinate system. The segments were defined between the reference heights below:

1. From 20 mm above the joint line to the joint line (20 mm–0).
2. From 10 mm above the joint line to the joint line (10 mm–0).
3. From 10 mm below the joint line to 10 mm above the joint line (−10 mm–10 mm).
4. From 10 mm below the joint line to the joint line (−10 mm–0).
5. From 20 mm below the joint line to the joint line (−20 mm–0).
6. From the widest intercondylar diameter to the joint line (0–WID).

As mentioned earlier, we aimed to compare the MRI-originated dataset with the graft diameter measured during surgery. ST and GT were harvested using a tendonstripper to detach the tendon at the level of musculotendinous transition. The remaining muscle tissue was removed from the tendons and the ST and GT were then bundled twice. The resulting four-stranded graft (STGT-graft) was then sewn together with a baseball stitch at both ends over a length of 3 cm each. The STGT graft diameter was measured using a graft sizing block (Arthrex Graft Sizing Block AR-1886, Arthrex, Munich, Germany) with holes of 0.5-mm increments. The graft diameter was measured by referring to the smallest hole that, could be passed by the graft with a tight fit but free passage.

### 2.3. Statistical Analysis

As the femoral bone and tendons were manually segmented by different researchers, we performed an intraclass correlation coefficient (ICC) using Cronbach’s alpha to assess the interrater reliability of measurements taken by the three observers. To identify any potential correlations between the cross-sectional area (CSA) and graft diameter measured during surgery, a Pearson correlation coefficient was calculated with a *p*-value < 0.05 being considered significant. IBM SPSS (Statistics for Macintosh, Version 29.0, IBM Corp., Armonk, NY, USA: IBM Corp.) served for statistical analyses. Due to the small sample size, post hoc power analyses were performed using G-power (Ver. 3.1.9.7, Erdfelder, Faul, & Buchner, 1996) indicating a statistical power of 0.54.

## 3. Results

Datasets from 32 patients were analyzed. An overview of their demographic data is illustrated in Table 1. The data were analyzed via a Shapiro–Wilk test, indicating a non-parametric distribution. Thus, a Wilcoxon signed-rank test was used to compare the male and female groups. Except for height and body weight, there was no significant statistical difference between groups.

An ICC then was calculated to account for potential observer-dependent differences in measuring the CSA. The ST, GT, and STGT CSA results were compared and evaluated according to Koo et al. [16]. The ICC values among the three observers were mostly between 0.8 to 0.9, reflecting good to excellent reliability (Table 2).

The STGT CSAs measured at six different levels were calculated for male and female patients by summarizing the CSAs of ST and GT, thus determining the STGT CSA. Values for the 12 female and 20 male patients as well as the STGT CSA in total are displayed in Table 3.

CSAs were higher in male than in female patients and also revealed differences in the segment revealing the largest CSA. However, this difference proved to be non-significant (*p* > 0.05).

In total, the largest cross-sectional area was documented in the levels 20 mm–0 and 0–WID. Over the course of the more caudally located levels, a steady reduction in the CSA was observed. This observation was the same in male patients but not to the same extent as in female patients.

The CSAs of STGT were then correlated with the diameter of the graft measured during surgery. Here, we also ran a subanalysis by gender. We detected a significant correlation between the CSA and determined graft diameter in the male population at all six levels (Table 4). The highest correlation in this group was seen at level 10 mm–0 with 0.537.

We observed correlations at every level in the female population as well. However, due to our small sample, these were significant in only 4 of 6 levels, except for levels −10 mm–0 and −20 mm–0 (Table 5). The strongest correlation in female patients was seen at the level WID–0 with 0.629.

We also conducted multiple linear regression for the mean graft diameter analyzing sex, body weight, BMI, and height as potentially independent determinants (Table 6). However, these results were not statistically significant.

The correlation between sex and the mean graft diameter was analyzed according to a general linear model. There was no statistically significant correlation between mean graft diameter and sex (*p* = 0.502) by considering BMI, body weight, and height as co-factors in the analysis.

Summarizing our results, we assessed the CSAs of all 32 STGTs as well, which revealed a significant correlation between the CSA of each level and the intraoperatively measured graft diameter. The results were significant for each evaluated level. The 10 mm–0 level revealed the strongest correlation, with a value of 0.552 (Table 7).

## 4. Discussion

The main findings of this study indicate that based on the three-dimensional model using MRI and segmentation, the specified CSA (cross-sectional area) of the STGT reveals a larger average area in men than in women. Furthermore, we observed a decrease in CSA from cranial to caudal levels among all patients. We also identified a clear correlation between the graft diameters measured intraoperatively and CSA values determined preoperatively in the MRI model. The strongest correlation appears at the 10 mm–0 level.

Relying on MRI data for pre-surgery calculations to assess whether the future ACL hamstring graft will be thick enough gives the surgeon significant advantages when planning the procedure [17,18,19]. Similar studies analyzed the correlation between the MRI CSA of the ST and GT and the combined STGT with the graft diameter determined intraoperatively at specific levels [7,20]. A direct comparison with our study’s three-dimensional model is, therefore, not possible. However, individual reference levels from other studies can be approximately related to the segments used in this study.

Hollnagel et al. investigated the predictive value of an MRI-determined CSA for the eventual diameter of the ACL graft in 64 patients undergoing ACL reconstruction with a hamstring graft [21]. MRI data from both 1.5 Tesla and 3 Tesla scanners were used from their clinic’s internal pool to ensure uniform image quality. The STGT cross-sectional area in their study was measured at both the joint line level and the level of the widest diameter of the medial femoral condyle under maximum magnification. An average value was then calculated from these two measurements. In the 1.5 Tesla group, the CSA of STGT showed a statistically significant correlation of 0.629 for this reference level. The reference level used in their study corresponds to the 0–WID segment in our study, although the correlation calculated in our study is lower.

However, the WID level is the most commonly used reference level for calculating the CSA of STGT [22,23,24,25,26].

Leitner et al. and Erquicia et al. examined the same level, and each study demonstrated a significant correlation [20,27]. The latter research group found the strongest correlation by conducting subgroup analyses based on the magnification used for MRI sequencing (2× magnification vs. 4× magnification). The 4× magnification group showed a stronger correlation (r = 0.86) than the 2× group (r = 0.52), raising the question of a possible influence of magnification on the correlation. Similar differences in the calculated correlation depending on magnification were also reported by Kremen et al. and Serino et al. [28,29]. The impact of magnification was found to be greater for the ST-CSA and GT-CSA than for the STGT-CSA [7,29].

The concept of correlating an average value derived from multiple measurements with a direct positional reference to the eventual graft diameter was investigated in the study by An et al. [30]. These authors initially used the reference level of the widest intercondylar diameter. Based on this slice, the cross-sectional area was also measured in the layers directly above and below under 4× magnification, allowing an average value to be calculated from those three measurements. A significant correlation between the graft diameter and average CSA values was calculated at r = 0.46. The reference levels in their study align approximately with the 0–WID segment in our study. However, our calculations yielded a much higher correlation for this segment, which supports the hypothesis that incorporating a greater number of cross-sectional area measurements results in a stronger correlation.

In general, it should be noted that the aforementioned studies standardized their MRI devices’ field strengths (1.5 Tesla and 3 Tesla) or divided their cohorts based on this parameter. While this approach ensures uniform image quality and spatial resolution, it fails to reflect the reality encountered in clinical practice. Patients often come to consultations bringing externally performed MRIs of varying quality. Our study specifically addressed this aspect by incorporating MRIs with different acquisition modalities within the analysis. This highlights the practical applicability of our study results and, together with the CSA calculations from 3D-segmented data, highlights this study’s innovative nature.

Looking ahead, the rapid advancement of artificial intelligence will enable even more precise and detailed analyses, further improving the quality of patient care.

### Limitation

The retrospective nature of this study represents a limitation as it represents an increased risk, particularly for selection bias and bias due to confounding. Furthermore, our small patient cohort of 32 patients diminishes the significance of our results and gives our findings a purely exploratory character with limited generalizability of results. Additionally, the heterogeneity of the imaging material used in our analysis resulted in differences in segmentation quality, which may negatively affect the assessment of CSA. Nevertheless, such heterogeneous imaging material reflects everyday clinical practice, which is why we chose this approach. However, standardizing MRI sequences and their slice protocols could be a way to improve the precision of segmentation and validate findings in a larger cohort.

## 5. Conclusions

MRI-based 3D segmentation and the resulting STGT CSA provide a valuable tool for the preoperative determination and estimation of the actual graft diameter of a quadrupled hamstring graft. The 10 mm–0 mm segment above the joint line emerged as the segment with the strongest correlation, making it a suitable reference for graft planning and graft options.

## Figures and Tables

**Table 1:** Demographic Overview.

	Female Mean ± SD	Male Mean ± SD	*p*-Value	Total Mean ± SD
Number of patients n	12	20		32
Age (years)	28.5 ± 13.7	27.4 ± 10.0	0.82	27.8 ± 11.4
Height (cm)	**163.1 ± 7.4**	**180.6 ± 6.2**	**0.001**	174.1 ± 10.9
Body weight (kg)	**66.3 ± 8.5**	**79.0 ± 8.1**	**0.001**	74.8 ± 10.5
BMI (kg/cm^2^)	25.1 ± 4.0	24.5 ± 2.6	0.96	24.7 ± 3.1
Graft diameter * (mm)	7.9 ± 0.5	8.2 ± 0.6	0.29	8.1 ± 0.5

SD standard deviation, * diameter measured during surgery, statistically significant results in bold.

**Table 2:** Inter-rater reliability.

Level	CSA ST	CSA GT	CSA STGT
20 mm	0.95	0.94	0.95
10 mm	0.96	0.93	0.96
Joint Line	0.84	0.95	0.92
−10 mm	0.95	0.91	0.95
−20 mm	0.89	0.60	0.85
WID	0.92	0.87	0.94

Values are expressed as the intraclass correlation coefficient for the three investigators using Cronbach’s alpha.

**Table 3:** Cross-sectional area STGT in mm^2^.

Segment	*n*	Female Mean ± SD	*n*	Male Mean ± SD	*p*-Value	*n*	Total Mean ± SD
20 mm–0	12	14.9 ± 5.7	20	17.9 ± 6.3	0.12	32	16.8 ± 6.1
10 mm–0	12	15.3 ± 6.2	20	17.7 ± 6.1	0.15	32	16.8 ± 6.1
−10 mm–10 mm	12	15.1 ± 6.3	20	17.3 ± 5.9	0.22	32	16.5 ± 6.1
−10 mm–0	12	15.2 ± 6.3	20	16.7 ± 6.0	0.31	32	16.2 ± 6.1
−20 mm–0	12	14.2 ± 6.2	20	16.2 ± 6.1	0.21	32	15.4 ± 6.1
0–WID	12	14.7 ± 5.2	20	18.1 ± 6.2	0.08	32	16.8 ± 6.0

SD standard deviation; values expressed as the sum of the cross-sectional areas of ST and GT (STGT).

**Table 4:** Pearson correlation between the intraoperatively determined graft diameter and the cross-sectional area of the STGT in male patients.

Segment	*n*	Correlation r	*p*-Value
20 mm–0	20	0.478	**0.033**
10 mm–0	20	0.537	**0.015**
−10 mm–10 mm	20	0.511	**0.021**
−10 mm–0	20	0.489	**0.029**
−20 mm–0	20	0.468	**0.038**
0–WID	20	0.487	**0.029**

Statistically significant results in bold.

**Table 5:** Pearson correlation between the intraoperatively determined graft diameter and the cross-sectional area of the STGT in female patients.

Segment	*n*	Correlation r	*p*-Value
20 mm–0	12	0.593	**0.042**
10 mm–0	12	0.603	**0.038**
−10 mm–10 mm	12	0.597	**0.040**
−10 mm–0	12	0.576	**0.050**
−20 mm–0	12	0.493	0.104
0–WID	12	0.629	**0.028**

Statistically significant results in bold.

**Table 6:** Multiple linear regression of BMI, height, and body weight using the graft diameter as analysis coefficient.

	Correlation Coefficient B	Standard Error	*p*-Value
BMI	0.404	0.247	0.113
Height	0.129	0.073	0.097
Body Weight	−0.135	0.085	0.122

**Table 7:** Pearson correlation between the intraoperatively determined graft diameter and the cross-sectional area of the STGT in all patients.

Segment	*n*	Correlation r	*p*-Value
20 mm–0	32	0.511	0.001
10 mm–0	32	0.552	0.001
−10 mm–10 mm	32	0.537	0.001
−10 mm–0	32	0.514	0.001
−20 mm–0	32	0.479	0.003
0–WID	32	0.528	0.001

## Data Availability

All data are within the manuscript; further inquiries can be directed to the corresponding author.
